# Impaction Bone Grafting Combined with Titanium Mesh for Acetabular Bone Defects Reconstruction in Total Hip Arthroplasty Revision: A Retrospective and Mini‐Review Study

**DOI:** 10.1111/os.13262

**Published:** 2022-04-20

**Authors:** Xiang Li, Bai‐qi Pan, Xiao‐yu Wu, Ming Fu, Wei‐ming Liao, Chu‐heng Wu, Pu‐yi Sheng

**Affiliations:** ^1^ Department of Orthopaedic The First Affiliated Hospital of Sun Yat‐Sen University Guangzhou China; ^2^ Guangdong Provincial Key Laboratory of Orthopaedics and Traumatology The First Affiliated Hospital of Sun Yat‐Sen University Guangzhou China; ^3^ Department of Joint Surgery The First Affiliated Hospital of Sun Yat‐Sen University Guangzhou China

**Keywords:** Acetabular defects, Bone mineral density, Impaction bone grafting, Revision, Total hip arthroplasty

## Abstract

**Objective:**

To investigate the application of impaction bone grafting (IBG) combined with Ti‐alloy mesh for acetabular bone defect reconstruction in total hip arthroplasty (THA) revision and follow up the clinical outcomes and imaging findings.

**Methods:**

The clinical and imaging data of patients who were admitted to our hospital from January 2000 to December 2020 and underwent acetabular bone defects reconstruction using IBG combined with titanium mesh were retrospectively analyzed. Preoperative and post‐revision Oxford and Harris scores, and post‐revision complications were evaluated. Radiographs were used to determine center of rotation (COR) of the hip joint, transparency line, bone graft fusion, and bone mineral density (BMD) around the hip joint.

**Results:**

Significant improvement was observed in both Oxford and Harris scores (*P* < 0.05). The radiographs taken at the last follow‐up examination showed no significant differences in the acetabulum COR, offsets, inclination angle, mean ratio of vertical value, and BMD analysis between the post‐revision side and contralateral side (*P* > 0.05). The follow‐up data showed restoration of the mesh implant and graft bone fusion.

**Conclusions:**

The application of IBG combined with titanium‐alloy mesh in revision THA patients with acetabular defects was found to provide satisfactory outcomes. However, large‐scale studies are still needed to further elucidate the long‐term outcomes.

## Graphical abstract

We investigate the application of impaction bone grafting (IBG) combined with titanium mesh for acetabular bone defects reconstruction in revision total hip arthroplasty (THA), and follow up the clinical outcomes and imaging findings. Post‐revision radiographs show the measurement of horizontal/vertical value of the acetabular implant, acetabulum offsets in a hip.

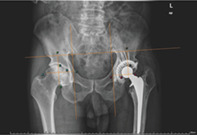

## Introduction

Joint arthroplasty has been proved to be a successful and cost‐effective treatment that can quickly restore joint function, relieve pain, and greatly improve patients' quality of life.^1^ The volume of primary total joint arthroplasty (TJA) operations has increased in recent years and is expected to continue to rise; the annual volume of primary total knee/hip arthroplasty (TKA/THA) are projected to reach over 3 million by 2030.^2^, ^3^, ^4^ Despite these encouraging trends, TJA carries risks associated with infections, aseptic prosthesis loosening, bone defect, *et cetera*. Among the post‐THA complications, prosthesis loosening, which is mainly caused by inflammatory osteolysis and stress shielding, is one of the major reasons for revision. Gradually, acetabular/femoral defects develop. These appear to be common problems in revision hip surgery, and most patients present with more serious acetabular bone defects when visiting a clinic.

Restoring bone mass is the key to reconstruct the acetabulum and ensure the stability of the acetabulum prosthesis.^5^ Therefore, management of acetabular defects is important to reconstruct the center of rotation (COR) of the hip and to provide initial stability of the revision prosthesis. Methods of reconstructing the acetabulum include placement of a jumbo cup, use of a high hip center, specialized roof and reconstruction rings, modular porous metal augments, bone voidllers, or bulk or morselized bone grafts.^6^, ^7^, ^8^, ^9^, ^10^, ^11^

Bone graft reconstruction is the only option that can restore bone remnants. This allograft may be in a structural form with uncertain results^12^ or in the form of an impacted graft.^13^, ^14^, ^15^ Quality and mechanical properties of the bone related to its mineral density are considered to be important predictors of TJA failure. A study reported that osteoporosis was an independent risk factor for prosthesis‐related complications following THA.^16^ A considerable portion of patients undergoing THA are those who have a higher risk of osteoporosis.^17^, ^18^ Recently, treatment with anti‐receptor activator of nuclear factor kappa‐B ligand (RANKL) monoclonal antibodies and bisphosphonates was found to be potentially effective to prevent THA revision in patients with osteoporosis.^19^, ^20^ Regular anti‐RANKL monoclonal antibody treatment prevents early periprosthetic bone loss after uncemented THA; however, the effect diminishes after discontinuation of treatment.^20^ These results indicate that despite a successful operation, postoperative monitoring and prevention of bone loss may also play critical roles in the entire succession of treatment for primary or revision patients, especially those with bone defects.

In an effort to reduce costs and improve survival rates for the increasing number of arthroplasty revision patients with acetabular defects, there has been an increasing focus on developing rapid protocols and strategies for the stability of the reconstructed acetabulum. Some reports have indicated that it may be both practical and effective to perform impaction bone grafting (IBG) combined with titanium‐alloy (Ti‐alloy) mesh for acetabular bone reconstruction. However, for types II and III acetabular defects, the long‐term outcome of IBG combined with Ti‐alloy mesh for acetabular reconstruction is still controversial.

In this study, we retrospectively analyzed clinical data, assessed radiographic images and acquired measurements of bone mineral density (BMD) in the periprosthetic complications of THA. The aims of our study were as follows: (i) to evaluate the outcome of prosthesis revision using IBG combined with Ti‐alloy mesh for acetabular reconstruction; (ii) to evaluate the risk of implant loosening as measured *via* BMD radiographical measurement; and (iii) to provide patients who have undergone THA with references for clinical selection of appropriate treatment methods.

## Methods

The study was approved by the institutional review board of the authors' affiliated institution. Informed consent was obtained from all patients. All staff and patients were blinded to the study. This study was approved by the Ethics Committee of the First Affiliated Hospital of Sun Yat‐Sen University ([2021]676). This was a retrospective study with follow‐ups from January 2000 to December 2020 conducted by a clinical team. Joint prosthesis loosening/infection and acetabular bone defect are the main indications for IBG combined with Ti‐alloy mesh in THA revision. According to the Paprosky classification, acetabular bone defects are divided into types I to III with different degrees of osteolysis and progressive bone loss involving the edges, which can further lead to the failure of the prosthesis and require suppression of bone grafting combined with Ti‐alloy mesh revision as treatment.^21^, ^22^

### Inclusion and Exclusion Criteria

We retrospectively reviewed 198 reports of patients who underwent consecutive revision THA. Patients were included based on the following criteria: (i) had revision surgery for acetabular prosthesis loosening; (ii) were found to have acetabular bone defect after revision surgery; (iii) had acetabular defect repaired by IBG combined with Ti‐alloy mesh; and (iv) had qualified follow‐up data.

The exclusion criteria were as follows: (i) fracture around the acetabular prosthesis caused by trauma due to loosening; (ii) dislocation of the acetabular prosthesis occurred due to its poor placement during the initial replacement; and (iii) patients who had other serious underlying diseases or mental function abnormalities, or who failed to cooperate with the diagnosis, treatment, and subsequent recovery. Using the above criteria, five patients were included. Demographic data and baseline information are detailed in Table 1.

**TABLE 1:** Patient demographics of all patients available for follow‐up

	AGE (sex)	BMI	Cause	Reconstructed bone type	Function follow‐up month	Radiographic follow‐up month	Compli‐cation	Smoker	Side
Case 1	88 (F)	29	APL	Allograft	75	24	n	n	R
Case 2	44 (M)	22	APL	Allograft	127	74	n	n	L
Case 3	58 (F)	24	APL	Allograft	136	73	n	n	R
Case 4	64 (M)	23	APL	Allograft	98	79	y	n	L
Case 5	56(F)	22	APL	Allograft	85	50	n	n	L

Abbreviation: APL, aseptic prosthesis loosening.

### Clinical and Surgical Management

The posterior lateral approach was used to expose the joint capsule. During the exposure process, the scars and adhesions around the joint were cleaned up. After the hip prosthesis was dislocated and the acetabulum prosthesis was removed, we took and mixed a suitable amount of ipsilateral iliac bone and allograft bone particles (particle diameter, 5–8 mm). Impaction particle bone grafts were used to fill supra‐acetabulum defects or the acetabulum top, anterior/posterior column, bottom or segmental wall bone defects. The integrity and support of the anterior and posterior columns and the acetabulum roof were reconstructed to change the mixed bone defect into a simple inclusive bone defect, and the normal structure of the acetabulum as far as possible. After repeated IBG was completed for every layer, the corresponding Ti‐alloy mesh cup was fixed using five screws of appropriate lengths on the acetabulum and the ischial bone in certain directions. After checking the stability of the Ti‐alloy cup to prevent uneven force on the acetabular cup, we blended the bone cement, pressed it into the Ti‐alloy mesh, and then loaded the corresponding type of plastic liner in at 40° abduction angle and 15° inclination. The acetabular cup was pressed and fixed completely to prevent the rotation of the acetabular mesh and ensure initial stability. After drainage removal, patients were mobilized under instructions from the clinician. Full weight bearing and full range of motion were allowed 4 weeks after surgery.

### Follow‐Up

All patients who agreed to be included in this trial completed radiological and functional follow‐up examinations (100%). The Harris hip score (HHS) and Oxford hip score (OHS) were used to evaluate the preoperative and post‐revision functions of the hip.

**Fig. 1:**
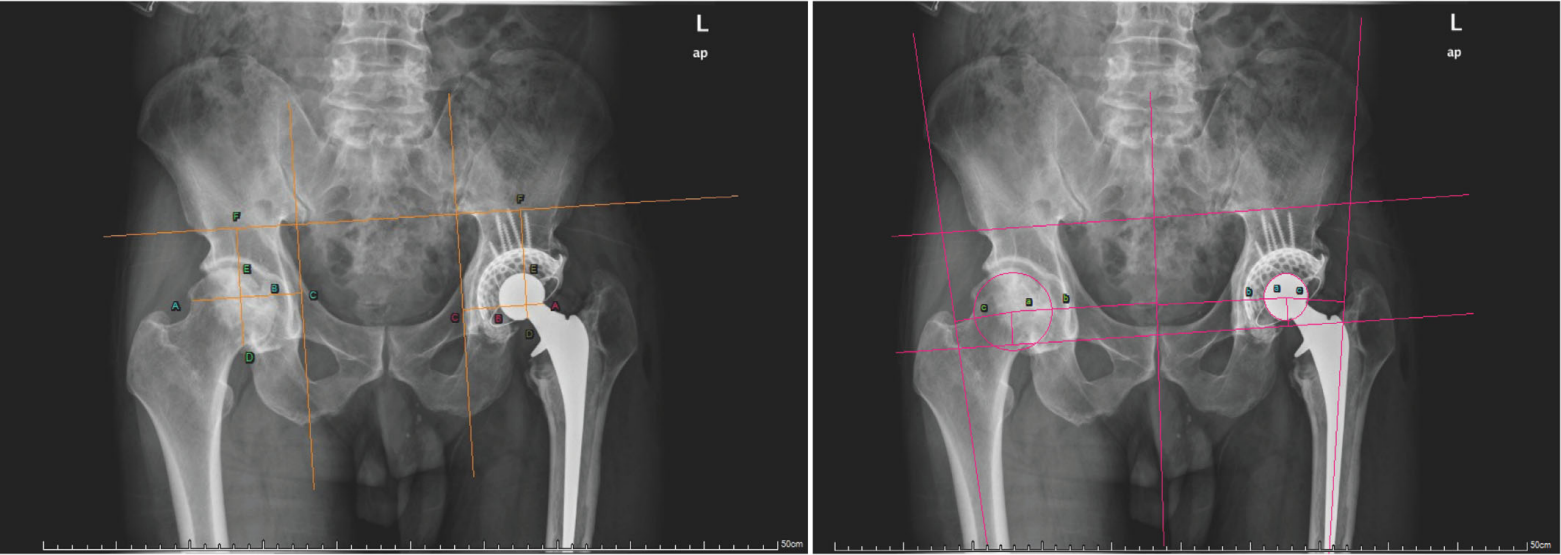
(A) Post‐revision radiographs showing the measurement of horizontal value (AC, BC), vertical value (DF, EF) of the acetabular implant, inclination angle of acetabulum (G); (B) Measurement of offsets in a hip (a: medial offset; b: ilioischial offset; c: femoral offset; d: center of rotation).

**Fig. 2:**
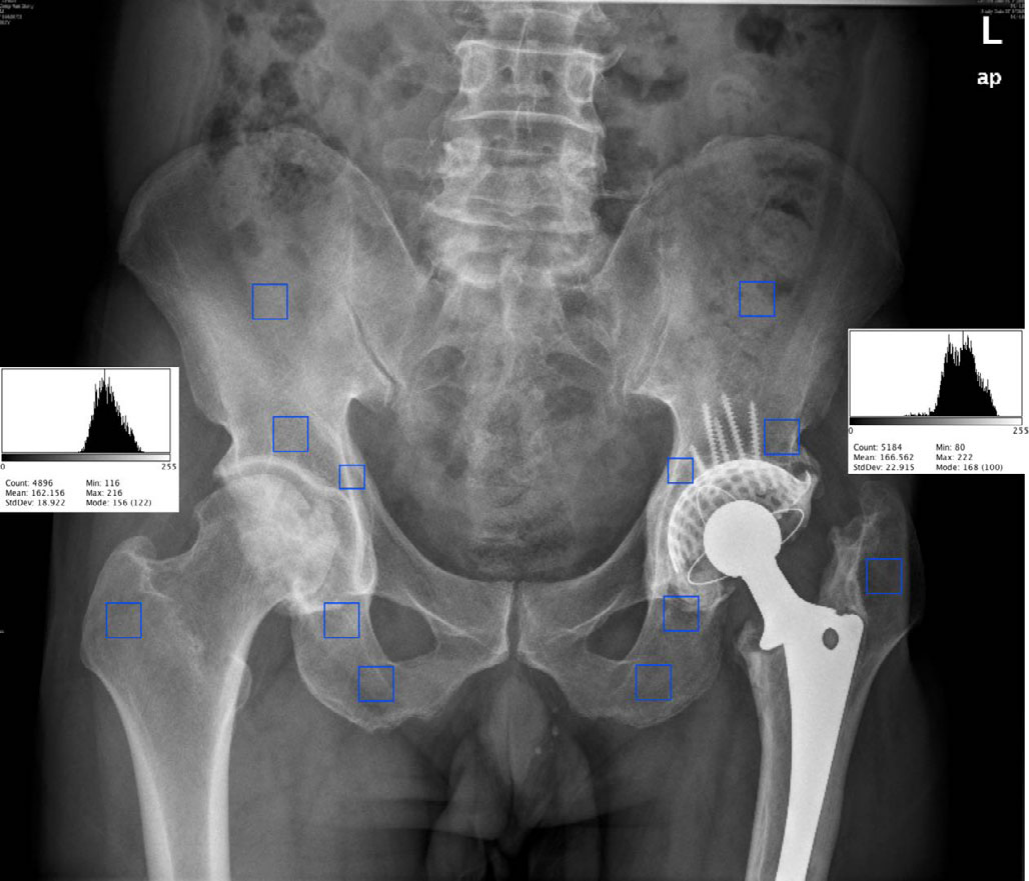
Region of interest (ROI) histogram analysis and ROIs of certain area. Diagram of Surgical Technique: We provide a manual sketch of the key procedures of the surgical technique.

Complications (pulmonary embolism, deep vein thrombosis, infection, pneumonia, dislocation), readmission rates (all causes), and mortality were included in the follow‐up data. Radiological follow‐up included post‐revision radiography or computed tomography scans. The evaluation of the radiographic outcomes of THA revision included analyzing horizontal and vertical migration of the prosthesis and measuring the acetabulum inclination angles^23^ (Fig. 1A). We also compared offset measurements including femoral offset, medial offset, ilioischial offset, and COR^15^ in both revision and contralateral sides of the hip (Fig. 1B). Digital image analysis was performed using a software program, ImageJ on regions of interest (ROIs) (Fig. 2). After histogram equalization to ensure standardization, gray‐scale levels were used with the metal density and air density as the maximum and minimum density references.^24^ The optical density was measured in the defined ROI over a 0–255 gray scale. The effect of bone grafting was evaluated by referring to Gross's standard,^25^ and the transparency line of the acetabular side was partitioned and described according to the method described by Delee.^26^

### Harris Hip Score Description

Harris hip score (HHS) was used for the assessment of the hip surgery results, with the intention to evaluate various hip disabilities and methods of treatment.^27^, ^28^ The score included pain, function, absence of deformity, and range of motion. The pain domain measures pain severity and its effect on activities and the need for pain medication. The function domain consists of daily activities (stair use, use of public transportation, sitting, and managing shoes and socks) and gait (limp, support needed, and walking distance). Deformity considers hip flexion, adduction, internal rotation, and extremity length discrepancy. Range of motion measures hip flexion, abduction, external and internal rotation, and adduction. The score has a maximum of 100 points (best possible outcome) covering pain (one item, 0–44 points), function (seven items, 0–47 points), absence of deformity (one item, 4 points), and range of motion (two items, 5 points).

### Oxford Hip Score Description

Oxford hip score(OHS) was developed to assess outcome after total hip replacement (THR) by measuring patients' perceptions in adjunction to surgery.^27^ The original version from 1996^29^ was updated in 2007 introducing a new scoring system.^30^ OHS assesses pain (six items) and function (six items) of the hip in relation to daily activities, such as walking, dressing, sleeping, *et cetera*, comprising 12 items with five categories of response without subscales. The original scoring ranged from 1–5 (best to worst) with a total score of 12–60 (least to most difficult).^29^ A new scoring was suggested in 2007 and supported by the original authors: 0–4 (worst to best) with overall scores ranging from 0 to 48, where 48 represents the best score.^30^

### Statistical Analyses

Statistical analyses were performed using IBM SPSS Statistics 19.0. Pre‐ and post‐revision (follow‐up) OHS and HHS were compared using the unpaired t‐test. The unpaired t‐test was also used to compare the radiological (COR, offsets, angle degrees, acetabulum value, and gray scale) differences between the post‐revision and contralateral sides of the hip. Statistical significance was reported as a *P* value ≤0.05.

## Results

### Demographic Data

From February 2000 to May 2020, a total of five patients who met the selection criteria were enrolled, which included two men and three women with an average age of 64.4 ± 15.95 years (range: 45–88 years). The average age of patients at the time of surgery was 55.4 ± 15.36 years (range: 34–76 years). The average body mass index (BMI) was 23.85 ± 2.88 kg/m^2^ (range: 22.03–28.89 kg/m^2^) (Table 1). All patients who agreed to be included in this study completed radiological and functional follow‐up examinations. The mean follow‐up length of functional examination was 103.2 ± 27.40 months (range: 75–136 months), while the radiographic follow‐up length was 69.75 ± 11.2 months (range: 50–79 months). Imaging tests showed Paprosky types II and III. The initial diagnosis for primary THA was femoral neck fracture (*n* = 2) and avascular necrosis of the femoral head (*n* = 3). The operation time was 180–980 min, with an average of 434.5 ± 320.73 min. The intraoperative blood loss was 600–3500 mL, with an average of 2180 ± 1293.06 mL. The duration of hospitalization was 15–46 days, with an average of 25.6 ± 12.86 days.

### Functional Assessment

**Fig. 3:**
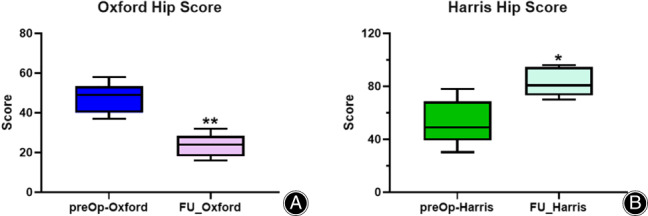
(A) Pre‐ versus post‐revision (Follow‐up) Oxford Hip Score; (B) Pre‐ versus post‐revision (Follow‐up) Harris Hip Score

The mean follow‐up length was 103.2 ± 27.40 months (range: 75–136 months). The average preoperative OHS and HHS was 47.2 ± 7.82 (range: 37–58) and 52.8 ± 17.44 (range: 30.4–78), respectively. The average OHS and HHS at the last follow‐up was 23.40 ± 5.99(16–32) and 83.30 ± 11.22(70–96), respectively; the differences were statistically significant (*P* < 0.01; Fig. 3). Preoperative limb shortening in two patients was greater than 3 cm and post‐revision limb shortening was <2 cm; the difference was statistically significant (*P* < 0. 01).

### Radiographic Assessment

Post‐revision hip joint rotation centers were all within the Renawat triangle.^31^ The offsets and height of COR of post‐revision radiologic follow‐up are shown in Fig. 1B. There was no significant difference between medial, femoral, and ilioischial offsets and height of COR in either hip.^15^ The median inclination angles of the post‐revision and contralateral sides were 41.60° ± 6.73° (range: 30.20–47°) and 39.16° ± 3.17° (range: 34.30–42.50°), respectively (Table 2), but the difference was not significant. The mean ratio of vertical and horizontal acetabular values was assessed considering radiographs obtained in the post‐revision follow‐up period, comparing the post‐revision and contralateral sides (Fig. 1A). Only the horizontal ratio showed a statistically significant difference (*P* = 0.02) (Table 2).

**TABLE 2:** Radiological results

Variable	Contralateral side	Post‐revision side	*t* value	*P* value
Horizontal value (ratio)				
Mean (standard deviation)	0.30 (0.07)	0.43 (0.06)	2.98	0.02
Minimum; maximum	0.23; 0.38	0.40; 0.55		
Vertical value (ratio)				
Mean (standard deviation)	0.49 (0.10)	0.56 (0.05)	1.45	0.18
Minimum; maximum	0.36; 0.59	0.51; 0.65		
Angle of inclination (deg)				
Mean (standard deviation)	39.16 (3.17)	41.60 (6.73)	−0.73	0.48
Median	40.00	44.80		
Minimum; maximum	34.30; 42.50	30.20; 47.00		

*P* < 0.05 indicate statistical significance.

The values (density average of the six ROIs) between the contralateral and operative sides showed no significant difference (106.23 ± 30.94 *vs* 101.18 ± 28.60, *P* = 0.51) (Table 3). The same counteracted areas and measurement methods were used in each case. We found no decrease in density from follow‐up data in the three acetabulum (top/supra/infra) ROIs individually, and also in the averaged ROI from the iliac crest and proximal femur.

**TABLE 3:** Clinical and radiological results

	Preoperative	Follow‐up	*t* value	*P* value
Oxford hip score (48)	47.2 ± 7.82	23.40 ± 5.98	5.40	<0.01
Harris hip score (100)	52.8 ± 17.44	83.30 ± 11.22	3.29	0.011

	Contralateral side	Post‐revision	*t* value	*p* value
Femoral offset (cm)	4.42 ± 0.32	4.35 ± 0.86	0.16	0.88
Medial offset (cm)	9.77 ± 0.44	9.46 ± 0.35	1.22	0.26
Ilioischial offset (cm)	4.06 ± 1.04	4.05 ± 0.78	0.21	0.98
Center of rotation (cm)	2.09 ± 0.45	2.62 ± 0.36	−2.03	0.076
Gray scale level of ROI	101.18 ± 28.60	106.23 ± 30.94	0.66	0.51

*P* < 0.05 indicate statistical significance.

According to Massin^26^ and Gross,^25^ only one acetabular prosthesis underwent loosening and bone graft resorption. In this patient, a 0.75 cm transparency line showed in the follow‐up radiography. There was no progressive transparency line at the acetabulum‐bone graft interface and no progressive transparency line around the screw in other patients. None of the acetabular prostheses had displacement, screw break, or joint dislocation. Analysis did not show acetabular prosthesis re‐revision for any reason as an end point.

### Post‐Revision Complications

Patients showed no related complications (pulmonary embolism, deep vein thrombosis, infection, pneumonia, joint dislocation), and there were no readmissions.

## Discussion

Post‐THA acetabular bone defect of varying degrees, is a common clinical problem. The factors leading to its occurrence include the following: prosthesis loosening (the main factor), debris of wear particles inducing osteolysis of the periprosthesis, poor preoperative bone condition, osteoporosis, *et cetera*. Therefore, acetabular defects must be adequately managed in successful revision surgery to ensure the stability of the prosthesis and the recovery of the function of the affected joint.

Many techniques have been developed for the reconstruction of acetabular defects, and researchers have reported that the technique of combining IBG with Ti‐alloy mesh shows promising functional and radiological outcomes when performed on THA patients with different types of acetabular bone loss, high short‐ to mid‐term survivorship, and low complication rates leading to re‐operation. IBG can fill the defect and increase the amount of bone in the acetabulum; support the Ti‐alloy mesh for bone graft fixation, which aids in varied direction of screw fixation and bone cement infiltration; reduce stress shelter and effectively restore anatomical structure of acetabulum; and disperse the pressure of the acetabulum load.^32^ The post‐revision clinical/functional subjective scores were significantly higher than those before surgery.^33^

### The Outcome of Prosthesis Revision with IBG and Ti‐Alloy Mesh

In this study, we retrospectively analyzed the clinical data and discussed the outcomes of prosthesis revision using IBG combined with Ti‐alloy mesh for acetabular reconstruction. There was a long‐term follow‐up period of 109 ± 27.86 months for the patients in this study. Although there are numerous similar postoperative patients, the cases with such a long follow‐up period are relatively rare.

To evaluate the results of hip surgery, OHS and HHS were used to compared post‐revision functional outcomes. A total HHS of <70 is considered a poor result; 70–80 is considered fair, 80–90 is good, and 90–100 is an excellent result. Our results showed that the average OHS from preoperative state to the last follow‐up was 47.2 ± 7.82 (37–58) and 23.40 ± 5.99 (16–32), respectively and the average HHS from preoperative state to the last follow‐up was 52.8 ± 17.44 (30.4–78) and 83.30 ± 11.22 (70–96), respectively. The average OHS decreased by 23.8 points and HHS increased by 30.5 points and the difference between these two hip scores was statistically significant (*P* < 0.01) (Fig. 3), showing good functional recovery. Similar increases in hip scores were reported by related studies.^15^, ^23^ The average last follow‐up HHS of 83.30 was in the ‘good result’ range. Even the lowest score was in the ‘fair result’ range. Preoperative limb shortening of two patients was greater than 3 cm, and post‐revision limb shortening was less than 2 cm; the difference was statistically significant (*P* < 0. 01).

Several methods, such as COR, offsets, Ranawat triangle, Mose's circle method, and inclination are considered as effective and useful perimeter methods to judge the radiographic outcomes of THA. According to literatures,^15^, ^34^ COR/inclination or angle/offset was found to indicate the mobility of the acetabular implant (mesh) surrounded by the impacted bone graft and may manifest insufficient combination of IBG that could deteriorate the reconstruction stability. However, satisfactory clinical outcomes were consistent with these radiographic results.^23^, ^31^ In our study, to obtain more specific and detailed information about the outcomes, we selected and combined the perimeter evaluation methods (rotation center migration, typical offsets, COR, inclination angles), performed them on the revision and contralateral sides, and analyzed the difference to judge the success of grafting and implant during the follow‐up. Our results found that the median inclination angles of the post‐revision and contralateral sides, including offsets (femoral, medial, and ilioischial), showed no statistically significant difference (Table 3). COR over 35 mm is recognized as a high hip COR.^35^, ^36^ There were no such cases. Only the horizontal ratio showed a statistical difference between the post‐revision and contralateral sides (0.3 ± 0.07 and 0.43 ± 0.06, *p* = 0.02) (Table 2). We suggest that this was because the size of the femoral head replacement was designed to be smaller than the real femoral head, causing the difference in the horizontal distance (Fig. 1). In our study, we chose the representative standard (Delee and Gross's standard) to discern aseptic prothesis loosening (APL) bone graft absorption/fusion. Only one case of a transparent line >4 mm was found, according to the above standard, which proved that the Ti‐alloy mesh played a protective role in the healing of pressure bone graft.

### Evaluation of Implant Loosening Risk via BMD Radiographical Measurement

After fixation and bone graft, some crucial factors determining cup fixation and stability have proven to be indicative of good outcomes with THA, including age‐related bone loss and size and location of acetabular defect.^37^ BMD is considered to be an important predictor of TJA failure, and osteoporosis/osteopenia patients with low BMD may have a higher risk of periprosthestic complication. However, it is difficult to perform dual energy X‐ray absorptiometry in every patient to test BMD in order to discern whether the patient has osteoporosis or osteopenia in every period during the follow‐up.

Therefore, we assessed BMD through a more feasible method of radiography to evaluate the risk of implant loosening. The average density value of the six representative ROIs between the contralateral and operative sides, including three acetabulum (top/supra/infra) and the iliac crest and proximal femur, were 106.23 ± 30.94 *vs* 101.18 ± 28.60, showing no significant difference (*P* = 0.51, *P* > 0.05) (Table 3). Our findings showed that the patients who underwent IBG combined with Ti‐alloy mesh to reconstruct acetabular bone defect had no statistically significant difference in gray scale between the revision and contralateral sides, which indicated that this surgical technique may cause progressive bone loss in THA revision patients. These results show that IBG with mesh may be an effective means of reconstruction for restoration of the rotation center and bone graft fusion.

A few drugs including bisphosphonates and anti‐RANKL monoclonal antibodies have been found to reduce periprosthetic bone loss after THA in patients with osteopenia and osteoporosis.^19^, ^20^ Bisphosphonates could help prevent the accelerated periprosthetic bone loss after THA in patients with osteopenia and osteoporosis.^19^ Bisphosphonates could help prevent the accelerated periprosthetic bone loss after THA in patients with osteopenia and BMD.^38^ Several clinical trials have shown the anti‐RANKL monoclonal antibody treatment to help prevent the postoperative complications by inhibiting early periprosthetic bone loss and helping in the repair around the femoral stem prosthesis.^39^, ^40^, ^41^ These effective treatment outcomes underline the importance of measuring BMD in THA patients.

### Review of the References of IBG Treatment Methods to the THA Patients

The above evidence reveals that the application of our method of BMD evaluation, which provides calculations that can help monitor the risks of postoperative complications and indications of using anti‐RANKL monoclonal antibodies or bisphosphonates, could lead to faster diagnosis and better prevention of TJA complications. We explored articles about the outcomes of impaction grafting technique performed as a treatment on THA revision patients with acetabular defects, more specifically combined with metal/Ti‐alloy mesh. We also provided the latest information of this widely used technique. Eleven articles with 433 cases were included (Table 4).^21^, ^42^, ^43^, ^44^, ^45^, ^46^, ^47^, ^48^, ^49^, ^50^, ^51^ Clinical outcome follow‐up was performed in 10 studies. HHS was used in nine of 11 articles; Merle d'Aubigne and Postel score was used in one article. Studies have shown that postoperative HHS is significantly improved compared with preoperative score. The average preoperative HHS was 40.175 and the postoperative HHS was 80.82 in 206 cases, with a statistically significant difference (*P* < 0.05).

**TABLE 4:** review on the articles about IBG combined with mesh applied on THA revision with acetabular defect

authors	Follow‐up (months, average)	Cases, materials	Complications	Clinical outcome	Radiographic outcome	BMD evaluation
Jin *et al*.^37^	85.2mo	24, Ti‐alloy mesh	1 APL	HHS, Preoperative 38(12 ‐ 56); Postoperative:86(81 ~ 92).	1 probable/define loosening; 1 radiological failure (re‐revision)	NR
Chen *et al*.^38^	61.2mo	22, Ti‐alloy mesh	1 sciatic nerve injury 2 PJI	HHS, Preoperative (43.75 ± 13.45); Postoperative: (85.33 ± 7.84)	Better height of hip rotation center; no migration; better distance between hip rotation center and the base of acetabulum	NR
Buttaro *et al*.^39^	36mo (24 ~ 56mo)	23, metal mesh	3 APL; 1 dislocation;	Merle D'Aubigne´‐Postel score, Preoperative: 7.4; Postoperative:16.2.	6 radiological failure	NR
Wang *et al*.^40^	22.4mo	19, metal mesh	1APL； 1PJI； 1 sciatic nerve injury	HHS, Preoperative:42.5(31 ‐ 56); Postoperative:88.6(82 ~ 96).	1 radiological failure	NR
Ye *et al*.^41^	78mo	19, Ti‐alloy mesh, scaffold	N/A	HHS, Preoperative:38.7 ± 9.6; Postoperative: 87.6 ± 7.8.	0 radiological failure	NR
Zhao *et al*.^42^	46.8mo	23, Ti‐alloy mesh,	N/A	HHS, Preoperative 38; Postoperative: 77.	3 radiological failure	NR
Lian *et al*.^43^	47mo	21, Ti‐alloy mesh	1APL	HHS, Preoperative 55.7; Postoperative: 92.9.	1 radiological failure	NR
Chen *et al*.^44^	48.6mo	42, Ti‐alloy mesh	1 superficial infection	HHS, Preoperative (22.25 ± 10.31); Postoperative: (85.85 ± 9.31).	N/A	NR
Lin *et al*.^45^	137mo	13, Ti‐alloy mesh	2APL	HHS, Preoperative 42.5(31 ‐ 56); Postoperative: 88.6(82 ‐ 96).	2 vertical migration	NR
Wadell *et al*.^46^	47mo (13 ~ 128)	21, metal mesh	1APL	HHS, Postoperative: 35.5(28 ‐ 40).	1 radiological failure	NR
García‐Rey *et al*.^47^	120 mo (60 ~ 204)	206, metal mesh	N/A	N/A	28 radiological failure	NR

HHS, Harris Hip Score; NR, not reported

Postoperative radiographic assessments demonstrated that the acetabular component was loose with minimal osteolysis or cup migration in 9.9% of the cases included in this analysis. The cumulative probability of APL after the revision was found in only 2.1% of the patients, in addition to periprosthetic joint infection or instability. Five patients in 43 cases who showed radiologically probable or definite loosening/failure underwent re‐revision surgery (11.6%). For patients with acetabular bone defects after THA revision, correct prosthesis and bone graft choices are necessary conditions for effective treatment. Moreover, IBG combined with Ti‐alloy mesh technique studied in the present study is an effective treatment method, but the indications have not yet been fully elucidated. During the follow‐up process after THA revision surgery, we learned that clinical and imaging examination failures occurred in various types of acetabular bone defects. It is therefore necessary to improve on the follow‐up to prevent related complications, especially APL, in a timely manner.Thus, our follow‐up parameters included BMD, COR, and offset. We found that BMD was not included in any of the reviewed articles. However, other studies reported that the lack of BMD may be a risk factor for THA complications, especially APL. According to our study, considering the high probability of the radiographical failure (9.9%) and the APL as the major complication (2.1%), it values the improvement on follow‐up and postoperative measurements with more comprehensive methods.

This study has some limitations. First, the small sample size and the lack of a control group. Second, the follow‐up did not obtain all the images and scores in every post‐revision period. Third, we lacked the preoperative BMD data of every patient. Finally, only retrospective trials investigating IBG with mesh for acetabular defects are available. A prospectively planned trial with a larger sample size should be conducted in the future.

### Conclusion

Both the functional and radiographic results of our study showed good outcomes of IBG with Ti‐alloy mesh for acetabular bone defect in THA revision. Further studies of higher quality and larger size are required to assess the optimal indications for IBG with mesh in comparison with other types of surgical reconstruction options.
